# Sex Differences in the Effect of Vitamin D on Fatigue in Palliative Cancer Care—A Post Hoc Analysis of the Randomized, Controlled Trial ‘Palliative-D’

**DOI:** 10.3390/cancers14030746

**Published:** 2022-01-31

**Authors:** Caritha Klasson, Maria Helde Frankling, Anna Warnqvist, Carina Sandberg, Marie Nordström, Carina Lundh-Hagelin, Linda Björkhem-Bergman

**Affiliations:** 1 Department of Neurobiology, Care Sciences and Society (NVS), Division of Clinical Geriatrics, Karolinska Institutet, Blickagången 16, Neo Floor 7, SE-141 83 Huddinge, Sweden; maria.helde.frankling@ki.se (M.H.F.); linda.bjorkhem-bergman@ki.se (L.B.-B.); 2 Theme Cancer, Thoracic Oncology Center, Karolinska University Hospital, Solna, SE-171 64 Stockholm, Sweden; 3 Department of Environmental Medicine, Division of Biostatistics, Karolinska Institutet, Nobels väg 13, SE-171 77 Stockholm, Sweden; anna.warnqvist@ki.se; 4 Palliative Medicine, Stockholms Sjukhem, Mariebergsgatan 22, SE-112 19 Stockholm, Sweden; Carina.sandberg@stockholmssjukhem.se (C.S.); Marie.Nordstrom@stockholmssjukhem.se (M.N.); 5 Department of Health Care Sciences, Ersta Sköndal Bräcke University College, SE-100 61 Stockholm, Sweden; carina.lundh-hagelin@esh.se; 6 Department of Neurobiology, Care Sciences and Society (NVS), Division of Care Science, Karolinska Institutet, Alfred Nobels Alle 23, SE-141 83 Huddinge, Sweden

**Keywords:** vitamin D, randomized clinical trial, palliative, cancer, fatigue, sex differences, Edmonton Symptom Assessment Scale (ESAS), European Organization for Research and Treatment of Cancer Quality of Life Questionnaire Core 15 for Palliative Care (EORTC QLQ-C15-PAL)

## Abstract

**Simple Summary:**

Previous studies have shown an association between low 25-hydroxyvitamin D levels and fatigue in cancer patients. In the recently published randomized, placebo-controlled, double-blind, multicenter trial ‘Palliative-D’, the correction of vitamin D deficiency reduced opioid use and fatigue in vitamin-D-deficient cancer patients admitted to palliative care. No subgroup analyses in women and men were made in the Palliative-D study. This post hoc analysis suggests that the positive effect of vitamin D supplementation on cancer-related fatigue may be more pronounced in men than in women. The vitamin-D-induced effect on fatigue could not be explained by reduced opioid doses among the vitamin-D-treated patients. Future studies focused on analyzing sex differences in the effect of vitamin D in palliative cancer care is needed before firm conclusions can be drawn.

**Abstract:**

In the randomized, placebo-controlled, double-blind trial ‘Palliative-D’, vitamin D treatment of 4000 IE/day for 12 weeks reduced opioid use and fatigue in vitamin-D-deficient cancer patients. In screening data from this trial, lower levels of vitamin D were associated with more fatigue in men but not in women. The aim of the present study was to investigate possible sex differences in the effect of vitamin D in patients with advanced cancer, with a specific focus on fatigue. A post hoc analysis of sex differences in patients completing the Palliative-D study (*n* = 150) was performed. Fatigue assessed with the Edmonton Symptom Assessment Scale (ESAS) was reduced in vitamin-D-treated men; −1.50 ESAS points (95%CI −2.57 to −0.43; *p* = 0.007) but not in women; −0.75 (95%CI −1.85 to 0.36; *p* = 0.18). Fatigue measured with EORTC QLQ-C15-PAL had a borderline significant effect in men (−0.33 (95%CI −0.67 to 0.03; *p* = 0.05)) but not in women (*p* = 0.55). The effect on fatigue measured with ESAS in men remained the same after adjustment for opioid doses (*p* = 0.01). In conclusion, the positive effect of the correction of vitamin D deficiency on fatigue may be more pronounced in men than in women. However, studies focused on analyzing sex differences in this context must be performed before firm conclusions can be drawn.

## 1. Introduction

Fatigue is a common and distressing symptom in patients with advanced or metastatic cancer that affects their quality of life (QoL) negatively [1,2,3]. A recent meta-analysis indicated that approximately 60% of patients with advanced cancer suffer from fatigue [4]. Several biological mechanisms for cancer-related fatigue have been proposed, with particular current interest in inflammation and immunity [5,6,7]. In addition, oncological treatments and opioid use can contribute to fatigue in cancer patients.

The treatment of fatigue in the palliative cancer setting remains a challenge due to the multifactorial nature of this symptom [1,8]. According to systematic meta-analyses, evidence-based management of cancer-related fatigue should focus on behavioral and psychological intervention [9], since pharmacological intervention has shown limited effect [8,10]. Cancer-related fatigue seems to be more pronounced in women than in men [4,11], especially at the end of life [12]. However, studies on sex differences in the etiology and treatment of fatigue are limited [13].

In the randomized, placebo-controlled, double-blind trial ‘Palliative-D’, we studied the effect of vitamin D supplementation versus placebo on opioid use, infections, fatigue, and QoL in cancer patients receiving palliative care [14]. In patients completing the study (*n* = 150), opioid use, measured as fentanyl ug/h, was significantly lower in the vitamin D group compared to placebo: −6.7 ug/h after 12 weeks (*p* < 0.05). Change in fatigue assessed with the Edmonton Symptom Assessment Scale (ESAS) was greater in the vitamin D treatment group compared to placebo, with less fatigue in the vitamin D group: −1.1 points (*p* < 0.01). No difference between groups was observed when fatigue was assessed with EORTC QLQ-C15-PAL, neither was there a difference in self-assessed QoL or infections between the groups [14]. To measure the vitamin D status of the patients before and after supplementation, 25-hydroxyvitamin D (25-OHD) was measured in plasma, according to established guidelines for vitamin D assessment [15,16]. Only patients with vitamin D deficiency, defined as 25-OHD < 50 nmol/L, were randomized to the study drug. The vitamin D treatment was safe and well-tolerated, despite the high doses used, and the frequency of adverse events were the same in both treatment arms.

The Palliative-D study was not powered for any subgroup analyses between men and women [17]. However, in the screening cohort (*n* = 530), an association between low 25-OHD levels and more severe fatigue was evident in men, while 25-OHD levels did not correlate to fatigue scores in women [18]. These findings encouraged further studies on possible sex differences in the effect of vitamin D.

Since pharmacological treatment options for fatigue in palliative care are limited, and the evidence bases for these treatments are weak [8,10], the reported positive effect of vitamin D is highly interesting and deserves further study.

The primary aim of the present study was to investigate possible differences in treatment effect of vitamin D between men and women with advanced cancer regarding self-assessed fatigue, by performing a post hoc analysis of the data from the Palliative-D study. The secondary aim was to study if the improvement in fatigue in the vitamin D group could solely be explained by reduced opioid use in this group.

## 2. Materials and Methods

‘Palliative-D’ was a randomized, double-blind, placebo-controlled trial conducted in three advanced palliative home care teams in the Stockholm Region. Between November 2017 and June 2020, 530 patients with advanced or metastatic cancer admitted to palliative care were screened for eligibility. Patients with any type of advanced or metastatic cancer with at least a three-month predicted remaining lifespan, no comorbidity or medication increasing the risk of developing hypercalcemia or kidney stones, 25-OHD ≤ 50 nmol/L, and taking at most 400 IU vitamin D/day were randomized to either vitamin D3 4000 IU/day (cholecalciferol) or placebo for 12 weeks. Detailed inclusion and exclusion criteria have been published previously [17]. The primary objective of the trial was to study the effect of vitamin D supplementation on pain, with opioid dose (fentanyl µg/h) as outcome measure. Secondary objectives were the effect of vitamin D supplementation on infections, fatigue, QoL, and 25-OHD. Outcome measures were assessed every four weeks during the intervention period. In total, 244 patients were randomized in the trial, 123 to intervention and 121 to placebo. Of these, 150 patients completed all 12 weeks, 67 in the intervention arm (33 women and 34 men) and 83 in the placebo group (43 women and 40 men). The analysis of 25-OHD was performed using chemiluminescence immunoassay (CLIA) with a LIAISON instrument (DiaSorin Inc, Stillwater, MN, USA) with a detection range of 7.5–175 nmol/L CV 2–5%, at Karolinska University Hospital Laboratory. The high dropout rate was due to clinical deterioration and death, and Kaplan–Meier analysis revealed no significant survival difference between groups [14].

### 2.1. Assessment of Fatigue

Edmonton Symptom Assessment System (ESAS) was used for self-assessment of fatigue at the screening visit and thereafter every four weeks during the intervention period, with final assessment after 12 weeks. ESAS is a symptom assessment instrument with ten items assessed on an eleven-point numeric rating scale [19,20]. For assessment of fatigue in the ‘Palliative-D’ study, the “tiredness question” was used with verbal anchors “no tiredness” at 0 and “worst possible tiredness” at 10. A change of one unit on the eleven-point scale is considered clinically meaningful [21,22]. Symptom intensity scored as 1–3 is considered mild, 4–6 moderate, and 7–10 severe [20,23,24,25]. Patients are asked to assess symptoms they experience at the moment when assessment is made. The revised version of ESAS, ESAS-r, has been translated and validated in Swedish in palliative care [26].

At screening and at end of study, after twelve weeks, we also assessed fatigue with EORTC QLQ-C15-PAL [27], a shortened version of EORTC QLQ-C30. In this instrument, developed for use in cancer patients in the palliative phase of their disease trajectory, fourteen symptom-items are assessed on a four-point verbal scale: “not at all” (1); “a little” (2); “quite a bit” (3); and “very much” (4) [27]. Assessment of global QoL is performed on a seven-digit numeric scale with the verbal anchors “very poor” (1) and “excellent” (7) [27]. Patients are asked to review their symptoms during the past week, i.e., this instrument covers a different time frame compared to ESAS. EORTC QLQ-C15-PAL has been translated and psychometrically validated in Swedish, and reference values from Swedish populations have been obtained [28,29,30]. In the ‘Palliative-D’ study we assessed fatigue using the single question (Q) “Were you tired?” (Q11) [14]. Thus, the same method was used in this study.

In addition, the combined score of Q11 and Q7 (weakness question) was also analyzed, since this score is recommended to assess fatigue according to the original manual of EORTC-QLQ-C15-PAL [25]. In this analysis, the mean between Q11 and Q7 was calculated for each timepoint and subject and used in the linear regression analysis.

### 2.2. Ethical Considerations

The study was conducted according to the declaration of Helsinki and approved by the Regional Ethical Committee (Dnr: 2017/405-31/1; date of approval 7 April 2017). Written informed consent was obtained from all participants before any study-related procedures were performed. The study was approved by the Swedish MDA, EudraCT: 2017-000268-14 and registered at Clinicaltrials.gov, NCT:03038516. All permits were obtained prior to study start.

### 2.3. Statistical Analysis

Both continuous and ordinal variables were summarized using median and interquartile range. To detect significant differences in the demographic data between the groups of men and women treated with vitamin D and placebo, Mann–Whitney test was used. The effect of vitamin D on the different endpoints from the Palliative-D study was performed using linear regression analysis including all participants that had completed the 12-week trial, i.e., the per-protocol population (PP-population). In the main analysis, adjustments were made for treatment arm and baseline values. Secondary models were estimated adjusting for age, 25-OHD levels at baseline, colectomy, and oncological treatment at inclusion (chemotherapy, hormonal therapy, target therapy, or no treatment). These factors were chosen since they may affect the effect and absorption of vitamin D, based on previous experiences from vitamin D supplementation studies [17,31,32,33].

The prespecified and previously published plan for analysis [17] did not include a plan for subgroup analyses, and the study was not designed to detect mean differences in outcome measures in subgroups. However, in this post hoc analysis, subgroup analyses of men and women were performed with the same strategy as the main analysis described above. In addition, a separate analysis excluding potentially hormone-dependent cancer types was performed.

To study if the effect on fatigue could be explained by the reduced opioid use in the vitamin D arm, an additional model adjusting for opioid doses after 12 weeks was constructed for fatigue measured with both ESAS and EORTC QLQ-C15-PAL.

All analyses were carried out in Stata 15 (StataCorp, 2017; Stata Statistical Software: Release 15; College Station, TX: StataCorp LLC) and in GraphPad Prism 9.0. Statistical significance was considered as *p* < 0.05.

## 3. Results

This post hoc analysis was based on the 150 patients that completed the Palliative-D study, the so-called per-protocol population (PP) of the original study. The flow chart with details of included and excluded patients during the study period is presented in the original publication [14]. In the vitamin-D-supplemented group (*n* = 67), 33 women and 34 men completed the twelve weeks of intervention. In the placebo group (*n* = 83), 43 women and 40 men reached the end of study. The baseline demographic data of the PP-population of men (*n* = 74) and women (*n* = 76) randomized to vitamin D and placebo are presented in Table 1.

There were no significant differences in the baseline demographics between men and women except for S-creatinine (*p* < 0.0001) (Table S1). However, the reference interval of S-creatinine differs between the sexes: men ˂ 100 µmol/L, women ˂ 90 µmo/L. Further, at baseline, women randomized to intervention used more antibiotics/month than women receiving placebo (*p* = 0.01) (Table S2). No significant differences regarding any baseline characteristic were detected in men between the treatment arms (Table S3). No missing data was reported except for one patient who did not assess question 15, QoL, in EORTC QLQ-C15-PAL.

### 3.1. Fatigue

**Figure 1:**
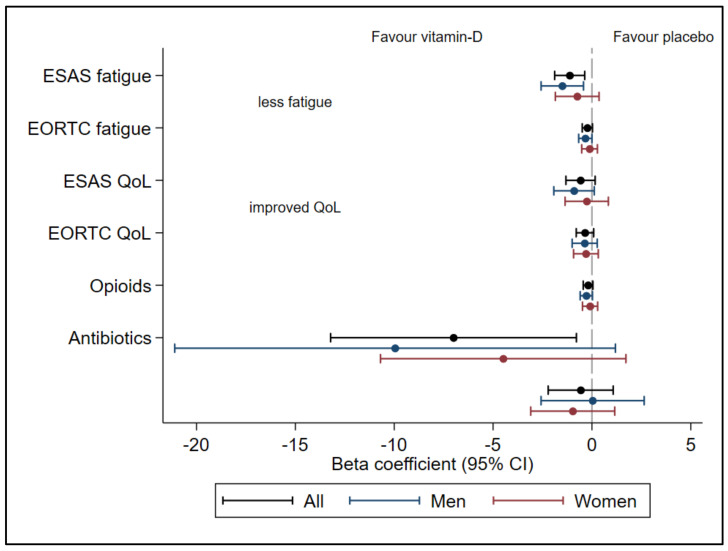
Outcomes in the Palliative-D study in men and women: forest plot with beta coefficient and 95% Confidence Interval (CI) over fatigue and quality of life (QoL) assessed with Edmonton Symptom Assessment Scale (ESAS) and EORTC-QLQ-PAL15 (EORTC), opioid doses and antibiotic consumption in the Palliative-D study. The analysis shows all 150 patients that completed the 12-week trial with vitamin D at 4000 IE/day (*n* = 67) or placebo (*n* = 83) and subgroup analysis of women (*n* = 33 and *n* = 43) and men (*n* = 34 and *n* = 40).

The positive effect on fatigue measured with ESAS was significant in men but not in women (Figure 1).

In men, the mean change was −1.50 ESAS points (95%CI −2.57 to −0.43; *p* = 0.007), and in women −0.75 (95%CI −1.85 to 0.36; *p* = 0.18). After adjustments for 25-OHD, age, colectomy, and oncological treatment, a positive effect remained for men (*p* = 0.01) but not for women (*p* = 0.23). Since sex hormones may affect fatigue, a separate analysis was performed where patients with potentially hormone-dependent cancer types where excluded (prostate, gynecological, and breast cancer). The results remained mainly unchanged in this analysis, with a significant effect in men (*p* = 0.006) but not in women (*p* = 0.63).

**Figure 2:**
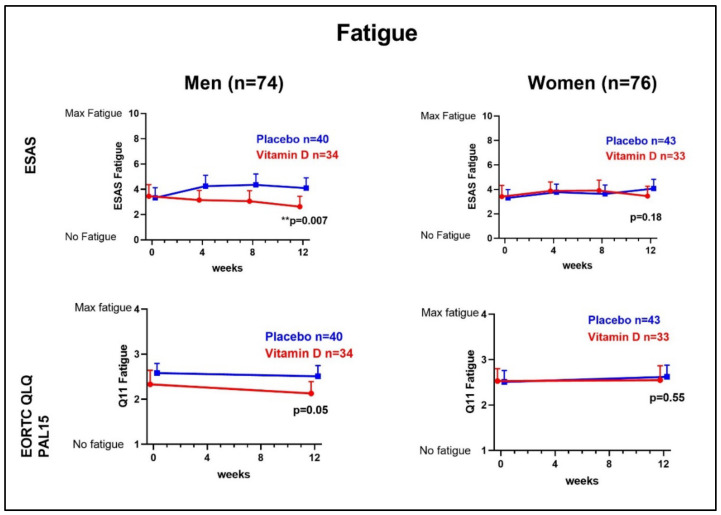
Fatigue in men and women: change in fatigue score measured with the “tiredness” question of Edmonton Symptom Assessment Scale (ESAS) and Question 11 of EORTC-QLQ-PAL15 throughout the study period in the Palliative-D study in men (*n* = 74) and women (*n* = 76). Points show mean of unadjusted raw data, +95% Confidence Interval (CI). The analysis is based on the 150 patients that completed the 12-week study period with vitamin D at 4000 IE/day (*n* = 67) and placebo (*n* = 83).

Using Q11 to assess fatigue with EORTC QLQ-C15-PAL did not render significant results when comparing supplemented and nonsupplemented patients in the entire cohort, nor in the subgroups of men and women (Figure 2).

However, in the adjusted model the effect of vitamin D on fatigue was significant in men, with a mean change of −0.38 (95%CI −0.74 to 0.03, *p* = 0.04). Similar results were obtained when using the combined score of Q7 and Q11 of EORTC QLQ-C15-PAL (Table 2).

### 3.2. Opioid-Induced Fatigue

In a separate analysis, adjustment was made for opioids to evaluate if the improved fatigue in the vitamin D group could be explained by the reduced opioid doses in this group. The effect on fatigue was only marginally affected by the change in opioid doses and was still significant in men (*p* = 0.01), but not in women (*p* = 0.18), as described in Table 3.

### 3.3. QoL, Opioids, and Antibiotics

There were no significant differences in the effect of vitamin D between men and women regarding QoL, opioid use, or antibiotics (Figure 1, Table 2). Interestingly, QoL in men assessed with ESAS indicated a nonsignificant improvement after vitamin D supplementation (*p* = 0.06), but this was not the case for EORTC QLQ-C15-PAL (*p* = 0.23). Women did not experience a significant effect on QoL (Table 2).

### 3.4. 25-Hydroxyvitamin D

**Figure 3:**
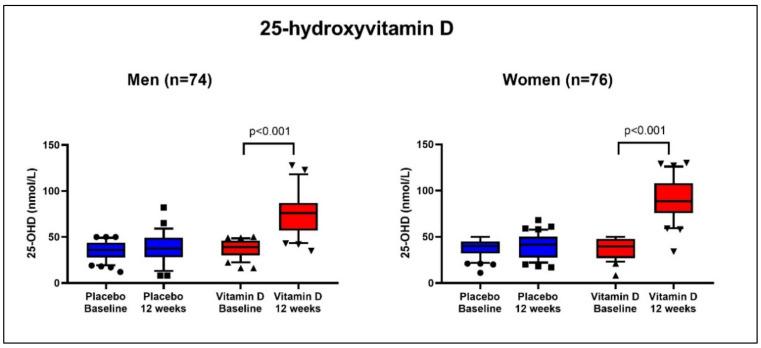
25-Hydroxyvitamin D levels: 25-hydroxyvitamin D (25-OHD) levels in women and men from the Palliative-D study at baseline and after 12 weeks of treatment with placebo or vitamin D at 4000 IE/day.

The change in 25-OHD levels in women and men throughout the study period is shown in Figure 3 and was significant both in men and women (*p* < 0.001). The increase in 25-OHD levels was more pronounced in women than in men; in women the increase was from an average of 38 to an average of 90 nmol/L, and in men from an average of 37 to an average of 77 nmol/L, thus implicating a reliable compliance in the entire cohort.

## 4. Discussion

In this post hoc analysis of patients with advanced cancer and vitamin D deficiency, men but not women experienced positive effects of vitamin D supplementation on fatigue. The observed effect of −1.5 ESAS points in men is considered to be clinically significant [21,22]. Interestingly, the effect on fatigue could not be solely explained by the reduced opioid doses in the vitamin D group. Thus, other mechanisms independent of opioid-induced fatigue must be involved.

A correlation between lower 25-OHD levels and increased fatigue in cancer patients has been established in some cohorts but not in all [18,34,35]. In addition, high 25-OHD has also been associated with higher health-related QoL (HRQoL) in one of these studies [34]. The result from the current study indicated a borderline significant effect on improved QoL in men but not in women. The result is in line with a study of colorectal cancer survivors, where higher 25-OHD levels were associated with improved QoL [34]. However, no sex differences were reported in that study. Vitamin D supplementation has shown positive results for self-reported fatigue in healthy individuals [36,37], in pooled data from two cohorts of patients with SLE [38], and in one out of two studies on patients with multiple sclerosis [39]. However, none of these studies reported any sex differences. To our knowledge, no interventional study with vitamin D to treat fatigue in cancer patients has been performed previously.

In the baseline data from all screened patients in the Palliative-D cohort, a significant association between vitamin D deficiency and fatigue was seen in men but not in women [18]. This is in line with a previous study showing a correlation between vitamin D levels and fatigue, where 77% of included patients were men [35]. Possible sex differences have also been shown in a study of curable colorectal cancer patients, where an association between improved fatigue, HRQoL, and higher vitamin D levels was observed only in men [34].

Two trials investigating the effect of vitamin D supplementation on fatigue in cancer patients are ongoing [40,41].

Several mechanistic explanations have been proposed regarding cancer-related fatigue, but cancer-induced inflammation and immunological processes seem to be involved [1,8]. Indeed, the severity of fatigue has been shown to be correlated with pro-inflammatory biomarkers [6]. Vitamin D is a steroid hormone with an important role in the immune system and has been shown to have anti-inflammatory properties [42,43]. In the case of vitamin D deficiency, the immune system favors a more inflammatory immune response involving Th1 and Th17 cells and increased levels of prostaglandins [44]. In contrast, if vitamin D levels are sufficient, the T-cell immune response shifts towards less inflammation and lower levels of inflammatory cytokines and prostaglandins [42,44]. Thus, the correction of vitamin D deficiency might reduce inflammation and subsequently affect fatigue [45]. We cannot offer a mechanistic explanation for the difference in effect between men and women observed in this study. However, one theory is that vitamin D affects male sex hormones, since previous studies have shown an association between low 25-OHD levels and hypogonadism in men [46]. In addition, an association between Vitamin D deficiency and muscle mass has been established in men but not women in a cross-sectional study of healthy patients ≥60 years [47]. Although there is conflicting data on the association between testosterone levels and 25-OHD levels [47,48], 25-OHD still seems to be involved in the modulation of sex hormone production [49]. Since low testosterone levels in men are associated with fatigue [50], there might be a link between low 25-OHD, low testosterone, and fatigue. However, in randomized, controlled trials, testosterone has not been shown to reduce fatigue in palliative care patients [10]. Additionally, since fatigue is affected by risk factors deriving from physical, psychological, social, and existential domains, there is a lack of information on how these factors are distributed between men and women and if they are interlinked and affected by vitamin D levels.

The strength of this study is that it is based on a randomized, placebo-controlled, double-blind multicenter study. It is also the first interventional study on sex differences in the effect of vitamin D on fatigue and HRQoL in palliative care.

The major limitation of this post hoc analysis is that the original study was not designed for subgroup analysis in women and men, constituting a risk of both type I and type II statistical errors. Thus, the results must be interpreted with caution and are only hypothesis-generating. In addition, the small numbers of patients who completed the study highlights the difficulties in performing trials in a palliative cohort with a high attrition rate due to death [51].

## 5. Conclusions

In conclusion, this post hoc analysis of the Palliative-D study showed that the correction of vitamin D deficiency reduced fatigue in men but not in women. However, since this is a post hoc analysis, future studies must be performed that are designed to study sex differences in the effects of vitamin D on fatigue in cancer patients before any firm conclusions can be drawn.

## Figures and Tables

**Table 1:** Demographic data at baseline of women and men completing the Palliative-D study: values show medians and interquartile range in parenthesis. Statistical analyses between the different subgroups are shown in Tables S1–S3.

Variable	Vitamin D		Placebo		
	Men (*n* = 34)	Women (*n* = 33)	Men (*n* = 40)		Women (*n* = 43)
Age, years	68 (60–75)	69 (59–75)	70 (64–73)		68 (59–73)
25-OHD, nmol/L	39 (30–46)	40 (27–48)	36 (28–44)		40 (32–45)
Fentanyl dose, µg/h	0 (0–25)	0 (0–37)	0 (0–22)		0 (0–12)
No. days on antibiotics/month	0 (0–0)	0 (0–3)	0 (0–4)		0 (0–0)
Albumin, g/L	32 (27–36)	32 (29–36)	31 (28–36)		32 (28–34)
Calcium, mmol/L	2.31 (2.21–2.38)	2.31 (2.23–2.42)	2.30 (2.22–2.36)		2.33 (2.26–2.41)
Creatinine, µmol/L	80 (68–97)	61 (55–77)	77 (67–96)		64 (55–77)
CRP, mg/L	4 (1–31)	7 (2–23)	8 (3–29)		5 (2–14)
Type of cancer, No. patients	… …		… …		
Brain	0	1	1	0	
Breast	0	4	0	10	
Upper gastrointestinal	10	6	13	6	
Lower gastrointestinal	12	9	10	10	
Gynecological	0	7	0	8	
Hematological	1	1	1	0	
Lung	4	5	6	6	
Prostate	7	0	7	0	
Sarcoma	0	0	0	3	
Urinary tract	0	0	3	0	
ESAS fatigue	4 (1–5)	3 (1–6)	3 (1–5)	3 (2–5)	
ESAS QoL	3 (1–5)	4 (2–6)	4 (2–5)	4 (2–5)	
EORTC QLQ-C15-PAL, Q11	2 (2–3)	2 (2–3)	3 (2–3)	2 (2–3)	
EORTC QLQ-C15-PAL, Q15	4 (3–6)	5 (4–5)	4 (3–5)	4 (3–5)	

In the placebo-treated male group there were 41 cancer diagnoses, due to one patient with 2 different types of cancer (prostate and upper gastrointestinal cancer). 25-OHD—S-25-hydroxyvitamin D; CRP—C-reactive protein; ESAS—Edmonton Symptom Assessment Scale (range 0–10); EORTC QLQ-C15-PAL—European Organization for Research and Treatment of Cancer Quality of Life Questionnaire C15 Palliative (fatigue Q 11 range 1–4, QoL Q 15 range 1–7); QoL—quality of life; Q—question.

**Table 2:** Effect of vitamin D at 4000 IU/day for 12 weeks on fatigue, quality of life (QoL), opioid doses, and antibiotic use in men and women using linear regression. The unadjusted model is adjusted for baseline values only. In the adjusted model, adjustments were made for 25-hydroxyvitamin D at baseline, age, colectomy, and oncological treatment.

	Men *n* = 74	
**Placebo *n* = 40,** **Vitamin D *n* = 34**	**Unadjusted Model**	**Adjusted Model**
**Variable**	**β (95% CI)** ***p*-Value**	**β (95% CI)** ***p*-Value**
Fatigue ESAS (0–10)	−1.50 (−2.57 to −0.43)	−1.47 (−2.59 to −0.35)
	** 0.007	* 0.01
Fatigue EORTC QLQ-C15-PAL Q11 (1–4)	−0.33 (−0.67 to 0.03)	−0.38 (−0.74 to 0.03)
	0.05	* 0.04
Fatigue EORTC QLQ-C15-PAL Q11 + Q7 (1–4)	−0.28 (−0.59 to 0.03)	−0.33 (−0.65 to −0.01)
	0.07	* 0.046
QoL ESAS (0–10)	−0.90 (−1.93 to 0.12)	−1.03 (−2.11 to 0.06)
	0.08	0.06
QoL EORTC QLQ-C15-PAL Q15 (1–7)	0.37 (−0.27 to 1.00) 0.25	0.41 (−0.26 to 1.08) 0.23
Opioid doses (µg fentanyl/h)	−9.96 (−21.10 to 1.19) 0.08	−5.67 (−17.34 to 5.99) 0.34
Antibiotics (days/month)	0.04 (−2.57 to 2.64) 0.98	0.99 (−1.68 to 3.67) 0.46
	**Women *n* = 76**	
**Placebo *n* = 43** **Vitamin D *n* = 33**	**Unadjusted Model**	**Adjusted Model**
Fatigue ESAS (0–10)	−0.75 (−1.85 to 0.36) 0.18	−0.71 (−1.87 to 0.45) 0.23
Fatigue EORTC QLQ-C15-PAL Q11 (1–4)	−0.12 (−0.52 to 0.28) 0.55	−0.15 (−0.56 to 0.27) 0.49
Fatigue EORTC QLQ-C15-PAL Q11 + Q7 (1–4)	−0.09 (−0.48 to 0.29) 0.62	−0.11 (−0.51 to 0.29) 0.57
QoL SAS (0–10)	−0.26 (−1.36 to 0.83) 0.63	−0.33 (−1.49 to 0.83) 0.57
QoL EORTC QLQ-C15-PAL Q15 (1–7)	0.30 (−0.32 to 0.93) 0.34	0.34 (−0.31 to 1.00) 0.30
Opioid doses (µg fentanyl/h)	−4.49 (−10.69 to 1.72) 0.15	−3.39 (−9.70 to 2.93) 0.29
Antibiotics (days/month)	−0.97 (−3.10 to 1.15) 0.37	−0.97 (3.17 to 1.23) 0.38

* *p* < 0.05, ** *p* < 0.01β—beta coefficient; CI—confidence interval; S-25-OHD—S-25-hydroxyvitamin D; ESAS—Edmonton Symptom Assessment Scale (range 0–10); EORTC QLQ-C15-PAL—European Organization for Research and Treatment of Cancer Quality of Life Questionnaire C15 Palliative (fatigue range 1–4, QoL range 1–7); ns—nonsignificant; QoL—quality of life; Q—question.

**Table 3:** Effect of vitamin D at 4000 IU/day for 12 weeks on fatigue, using linear regression. Unadjusted model is adjusted for baseline values only. The adjusted model is adjusted for opioid doses after 12 weeks, since these doses were reduced in the vitamin D group.

	Men *n* = 74	
**Placebo *n* = 40,** **Vitamin D *n* = 34**	**Unadjusted Model**	**Opioid-Adjusted Model**
**Variable**	**β (95% CI)** ***p*-Value**	**β (95% CI)** ***p*-Value**
Fatigue ESAS (0–10)	−1.50 (−2.57 to −0.43)	−1.42 (−2.51 to −0.34)
	** 0.007	* 0.01
Fatigue EORTC QLQ-C15-PAL Q11 (1–4)	−0.33 (−0.67 to 0.03) 0.05	−0.30 (−0.64 to 0.03) 0.08
	
Fatigue EORTC QLQ-C15-PAL Q11 + Q7 (1–4)	−0.28 (−0.59 to 0.03) 0.07	−0.26 (−0.57 to 0.05) 0.10
	
	**Women *n* = 76**	
**Placebo *n* = 43** **Vitamin D *n* = 33**	**Unadjusted Model**	**Opioid-Adjusted Model**
Fatigue ESAS (0–10)	−0.75 (−1.85 to 0.36) 0.18	−0.75 (−1.86 to 0.36) 0.18
Fatigue EORTC QLQ-C15-PAL Q11 (1–4)	−0.12 (−0.52 to 0.28) 0.55	−0.12 (−0.52 to 0.28) 0.56
Fatigue EORTC QLQ-C15-PALQ11 + Q7 (1–4)	−0.09 (−0.48 to 0.29) 0.62	−0.09 (−0.48 to 0.29) 0.62

* *p* < 0.05, ** *p* < 0.01, β—beta coefficient; CI—confidence interval; ESAS—Edmonton Symptom Assessment Scale (range 0–10); EORTC QLQ-C15-PAL—European Organization for Research and Treatment of Cancer Quality of Life Questionnaire C15 Palliative (fatigue range 1–4).

## Data Availability

The raw data are available from the corresponding author upon reasonable request.

## Supplementary Materials

The following are available online at https://www.mdpi.com/article/10.3390/cancers14030746/s1, Table S1: Baseline demographics of all men and women completing the Palliative-D study regardless of treatment group, Table S2: Baseline characteristics of women completing the Palliative-D study, split by treatment group of vitamin D or placebo, Table S3: Baseline characteristics of men completing the Palliative-D study, split by treatment group of vitamin D or placebo.
