# Supplementary material for: Sex Differences in the Effect of Vitamin D on Fatigue in Palliative Cancer Care—A Post Hoc Analysis of the Randomized, Controlled Trial ‘Palliative-D’
Source: Cancers (Basel). 2022 Jan 31;14(3):746. doi: 10.3390/cancers14030746 (PMC8833647; doi:10.3390/cancers14030746)
Supplement: Supplementary file 1 [file cancers-14-00746-s001.zip › cancers-1516375-supplementary.pdf]

Table S1. Baseline demographics of all men and women completing the Palliative-D study regardless of treatment group. For statistical significance, Mann-Whitney test was used.

Variable	All men (n = 74) Median (IQR)	All women (n = 76) Median (IQR)	p-value
Age, years	68 (64–74)	68 (58–73)	0.60
25-OHD, nmol/L	37 (30–45)	40 (31–46)	0.26
Fentanyl dose, ug/h	0 (0–25)	0 (0–12)	0.59
No. days on antibiotics/month	0 (0–2)	0 (0–0)	0.09
Albumin, g/L	31 (27–36)	32 (28–35)	0.57
Calcium, mmol/L	2.30 (2.22–2.36)	2.32 (2.25–2.42)	0.11
Creatinine, µmol/L	79 (68–95)	64 (55–77)	***<0.0001
CRP, mg/L	7 (2–29)	7 (2–20)	0.47
ESAS fatigue	3 (1–5)	3 (1–5)	0.91
ESAS QoL	3 (2–5)	4 (2–5)	0.56
EORTC QLQ-C15-PAL, Q11 fatigue	2 (2–3)	2 (2–3)	0.96
EORTC QLQ-C15-PAL Q15 QoL	4 (3–5)	4 (3–5)	0.45

*p < 0.05, **p < 0.01, ***p < 0.001, S-25-OHD: S-25-hydroxyvitamin D, CRP: C-Reactive Protein, ESAS: Edmonton Symptom Assessment Scale (range 0-10), EORTC QLQ-C15-PAL: European Organization for Research and Treatment of Cancer Quality of Life Questionnaire C15 Palliative (fatigue Q 11 range 1-4, QoL Q 15 range 1-7), QoL: Quality of Life Q: Question. Creatinine reference interval ≥18 years: men <100µmol/L, women <90µmo/L.

Table S2. Baseline characteristics of women completing the Palliative-D study, split by treatment group of vitamin D or placebo.

Variable	Vitamin D	Placebo	
	**Women (n = 33)** Median (IQR)	**Women (n = 43)** Median (IQR)	**p-value**
Age, years	69 (59–75)	68 (59–73)	0.71
25-OHD, nmol/L	40 (27–48)	40 (32–45)	0.85
Fentanyl dose, ug/h	0 (0–37)	0 (0–12)	0.26
No. days on antibiotics/month	0 (0–3)	0 (0–0)	*0.01
Albumin, g/L	32 (29–36)	32 (28–34)	0.25
Calcium, mmol/L	2.31 (2.23–2.42)	2.33 (2.26–2.41)	0.45
Creatinine, µmol/L	61 (55–77)	64 (55–77)	>0.99
CRP, mg/L	7 (2–23)	5 (2–14)	0.82
ESAS fatigue	3 (1–6)	3 (2–5)	0.83
ESAS QoL	4 (2–6)	4 (2–5)	0.84
EORTC QLQ-C15-PAL, Q11 fatigue	2 (2–3)	2 (2–3)	0.90
EORTC QLQ-C15-PAL Q15 QoL	5 (4–5)	4 (3–5)	0.25

*p < 0.05, **p < 0.01, ***p < 0.001, S-25-OHD: S-25-hydroxyvitamin D, CRP: C-Reactive Protein, ESAS: Edmonton Symptom Assessment Scale (range 0-10), EORTC QLQ-C15-PAL: European Organization for Research and Treatment of Cancer Quality of Life Questionnaire C15 Palliative (fatigue Q 11 range 1-4, QoL Q 15 range 1-7), QoL: Quality of Life Q: Question.

Table S3. Baseline characteristics of men completing the Palliative-D study, split by treatment group of vitamin D or placebo.

Variable	Vitamin D	Placebo	
	**Men (n = 34)** Median (IQR)	**Men (n = 40)** Median (IQR)	**p-value**
Age, years	68 (60–75)	70 (64–73)	0.73
25-OHD, nmol/L	39 (30–46)	36 (28–44)	0.37
Fentanyl dose, ug/h	0 (0–25)	0 (0–22)	0.98
No. days on antibiotics/month	0 (0–0)	0 (0–4)	0.33
Albumin, g/L	32 (27–36)	31 (28–36)	0.95
Calcium, mmol/L	2.31 (2.21–2.38)	2.30 (2.22–2.36)	0.94
Creatinine, µmol/L	80 (68–97)	77 (67–96)	0.80
CRP, mg/L	4 (1–31)	8 (3–29)	0.45
ESAS fatigue	4 (1–5)	3 (1–5)	0.76
ESAS QoL	3 (1–5)	4 (2–5)	0.47
EORTC QLQ-C15-PAL, Q11 fatigue	2 (2–3)	3 (2–3)	0.20
EORTC QLQ-C15-PAL Q15 QoL	4 (3–6)	4 (3–5)	0.61

*$p < 0.05$, **$p < 0.01$, ***$p < 0.001$, S-25-OHD: S-25-hydroxyvitamin D, CRP: C-Reactive Protein, ESAS: Edmonton Symptom Assessment Scale (range 0-10), EORTC QLQ-C15-PAL: European Organization for Research and Treatment of Cancer Quality of Life Questionnaire C15 Palliative (fatigue Q 11 range 1-4, QoL Q 15 range 1-7), QoL: Quality of Life Q: Question.
